# Optical characterization of Tm^3+^ doped Bi_2_O_3_-GeO_2_-Ga_2_O_3_ glasses in absence and presence of BaF_2_

**DOI:** 10.1038/srep31207

**Published:** 2016-08-10

**Authors:** Kexuan Han, Peng Zhang, Shunbin Wang, Yanyan Guo, Dechun Zhou, Fengxia Yu

**Affiliations:** 1 Changchun University of Science and Technology, 7089 Weixing Road, Changchun, Jilin, 130022, P. R. China; 2 Jilin University, Changchun, Jilin, 130012, P. R. China

## Abstract

In this paper, Two new Bi_2_O_3_-GeO_2_-Ga_2_O_3_ glasses (one presence of BaF_2_) doped with 1mol% Tm_2_O_3_ were prepared by melt-quenching technique. Differential thermal analysis (DTA), the absorption, Raman, IR spectra and fluorescence spectra were measured. The Judd–Ofelt intensity parameters, emission cross section, absorption cross section, and gain coefficient of Tm^3+^ ions were comparatively investigated. After the BaF_2_ introduced, the glass showed a better thermal stability, lower phonon energy and weaker OH^−^ absorption coefficient, meanwhile, a larger ~1.8 μm emission cross section *σ*_*em*_ (7.56 × 10^−21^ cm^2^) and a longer fluorescence lifetime *τ*_*mea*_ (2.25 ms) corresponding to the Tm^3+^: ^4^F_3_ → ^3^H_6_ transition were obtained, which is due to the addition of fluoride in glass could reduce the quenching rate of hydroxyls and raise the cross-relaxation (^3^H_6_ + ^3^H_4_ → ^3^F_4_ + ^3^F_4_) rate. Our results suggest that the Tm^3+^ doped Bi_2_O_3_-GeO_2_-Ga_2_O_3_ glass with BaF_2_ might be potential to the application in efficient ~1.8 μm lasers system.

Over the past decade, Tm^3+^-doped fiber lasers have attracted growing attention in numerous areas owing to their very broad transition linewidth over ~1.7 to 2.1 μm wavelength^1^,^2^,^3^,^4^. As we know, near-infrared lasers at the eye-safe 2 μm region have many potential applications in medicine, remote sensing, and atmospheric pollutant monitoring^4^,^5^. Recently, the long-wavelength window around 1700 nm has attracted attention for OCT imaging^6^. Wavelengths near ~1720 nm are of interest for targeting fat/lipid-rich tissues due to the high absorption coefficient of human fat and low water scattering and absorption^7^. Nicholas G. Horton. *et al*. were put forward expectations that a wavelength-tunable source that covers the entire “low attenuation” spectral window from 1650 to 1850 nm can be obtained, which will further increase the number of accessible fluorophores and fluorescent proteins for Three-photon fluorescence microscopy (3PM) in the 1700 nm spectral window^8^. In addition, they can operate as pump sources for achieving 3.0~5.0 μm mid-infrared fiber lasers output at room-temperature, for national defense and commercial applications^9^,^10^. A typical work on Tm^3+^-Tb^3+^ co-doped tunable fiber ring laser for 1716 nm lasing was pumped by a 1.21 μm laser diode^11^. Another type of Tm-doped silica fiber laser with narrow-linewidth and output wavelength near 1750 nm has been reported, by using a 1550 nm Er-doped fiber laser pump source and a volume Bragg grating (VBG)^12^.

Tm^3+^ is a better solution to ~2 μm emissions because of its absorption band near 808 nm matching well with commercially available and high power laser diode^13^. Due to the cross-relaxation (^3^H_6_ + ^3^H_4_ → ^3^F_4_ + ^3^F_4_) process between Tm^3+^ ions, the ideal quantum efficiency of Tm^3+^: ^3^F_4_ can reach 200%^14^,^15^. To date, in order to get powerful infrared emissions from Tm^3+^ ions, various kinds of glass hosts have been investigated including silicate^16^, tellurite^17^, germanate^18^, and fluorophosphates^19^ glasses. Yin-Wen Lee, etc. reported an 18-dB 2013-nm amplifier which was demonstrated in a 50-cm 7 wt% Tm^3+^-doped double-clad silicate fiber^20^. Xin Wen, etc. reported a multilongitudinal-mode fiber laser at 1.95 μm has also been achieved in a 10 cm long as-drawn active fiber, yielding a maximum laser output power of 165 mW and a slope efficiency of 17%^21^. Zhi-Xu Jia reported a supercontinuum generation in Tm^3+^ doped tellurite microstructured fibers pumped by a 1.56  μm femtosecond fiber laser^22^. However, few researches have been paid on the bismuth germanate glass and fiber.

Among the oxide glasses, the bismuthate glass has a lower phonon energy (~440 cm^−1^)^23^,^24^ compared with silicate (~1000 cm^−1^)^16^, germanate (~900 cm^−1^)^13^ and tellurite (~750 cm^−1^)^17^ glasses, which is very useful to enhance the luminescence quantum efficiency^23^ of Tm^3+^ ions and reduce the multiphonon relaxation^24^. In addition, compared with silicate and other heavy metal oxide glasses, the bismuthate glass possesses many other material advantages such as easy preparation process, low melting temperature, large rare-earth solubility^21^, high refractive index (~2.1)^25^ and wide transparency window^26^, make bismuthate glass particularly promising for fiber amplifiers and infrared fiber lasers.

The OH^−^ groups may quench ^3^F_4_ → ^3^H_6_ emissions of Tm^3+^ ions and reduce emission efficiency^5^. But hydroxyl and the fluorine ions are isoelectronic and their ionic size was similar; hydroxyl ions could easily be removed by fluoride during melting^27^. Therefore, 1 mol% Tm^3+^-doped bismuth-germanium-gallate glasses in absence and presence of BaF_2_ were studied for ~1.8 μm emission.

## Experimental

Molar composition of 36Bi_2_O_3_− 29GeO_2_− 25Ga_2_O_3_− 10Na_2_O− 1Tm_2_O_3_ (BGN) and 36Bi_2_O_3_− 29GeO_2_− 25Ga_2_O_3_− 10BaF_2_− 1Tm_2_O_3_ (BGF) glasses were fabricated by conventional melting-quenching method in an alumina crucible at 1200 °C under oxygen atmosphere respectively. The glass samples were formed by casting molding and finally annealed at 480 °C for 3 h to remove thermal strains. Samples were cut and polished to 10 × 10 × 2 mm^3^ for property measurements.

Differential thermal analysis (DTA) was performed using a SETARAM TAG24 analyser, for characteristic temperatures (the temperature of glass transition *T*_*g*_, temperature of onset crystallization *T*_*x*_ and temperature of peak crystallization *T*_*p*_). Density and refractive index of samples was obtained by Archimedes method and spectroscopic ellipsometer method, respectively. The absorption spectrum was recorded using a spectrophotometer (Perkin Elmer Lambda9). The near-infrared emission spectra and luminescence lifetime were measured by FLSP920 (Edinburgh instruments Ltd., UK) under 808 nm laser diode pumped. Raman spectra were monitored with a FT Raman spectrophotometer (Nicolet Module). All measurements were carried out at room temperature.

## Results and Discussions

### Thermal property

**Figure 1:**
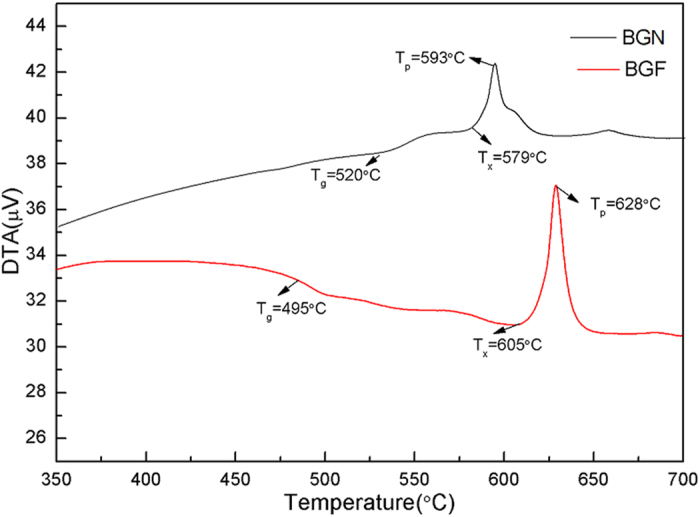
DTA curves of BGN and BGF glasses doped with 1 mol% Tm_2_O_3_ at the heating rate of 10 K/min.

Figure 1 shows the DTA curve of the studied glass, and the values of *T*_*g*_, *T*_*x*_ and *T*_*p*_ in Tm^3+^-doped BGN and BGF samples are indicated. The difference between the glass transition temperature *T*_*g*_ and the onset crystallization temperature *T*_*x*_, *ΔT* = *T*_*x*_ − *T*_*g*_, has been frequently used as a rough estimate of glass formation ability or glass thermal stability. It can be seen that the values of *T*_*g*_ is decreased from 520 °C to 495 °C as the Na_2_O is replaced by BaF_2_ in BGF glass. However, it is still higher than of fluoride^28^, tellurite^29^ glasses, this results show that the glasses have good thermal shock resistance performance under the condition of high power pump. Generally, the *ΔT* of the glass sample should be higher than 100 °C to obtain a better thermal stability and to avoid crystallization during the optical fiber drawing process^30^,^31^. After the addition of BaF_2_, the thermal stability (*ΔT*) of Bi_2_O_3_-GeO_2_-Ga_2_O_3_ glass is increased quite significantly. The value of *ΔT* for BGF sample is 110 °C,which is higher than of BGN (59 °C), indicating that the BGF sample has better thermal stability against crystallization for ~1.8 μm emission.

### Absorption and IR transmittance spectra

**Figure 2:**
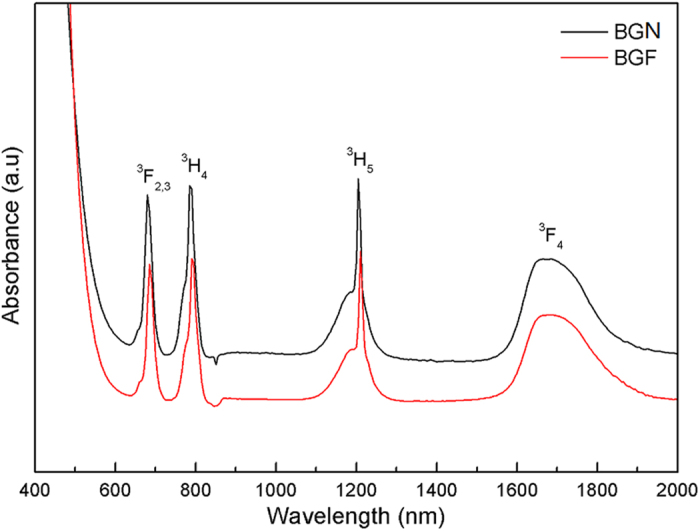
Room temperature absorption spectra in the range from 400 to 2000 nm of the BGN and BGF glass samples doped with 1 mol% of Tm_2_O_3_.

**Figure 3:**
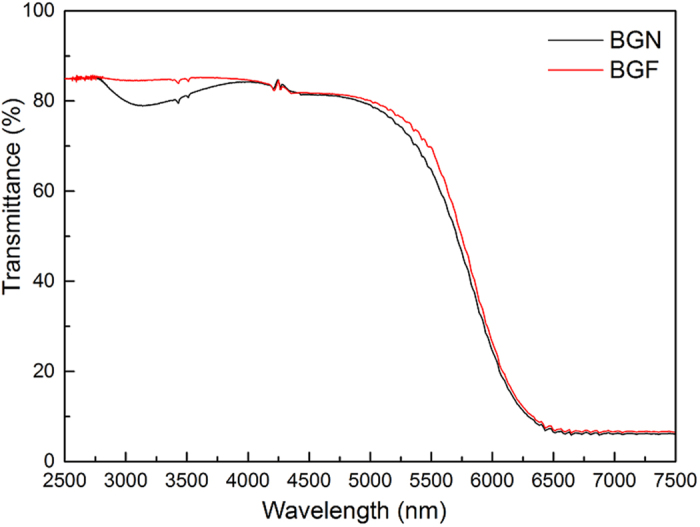
Infrared transmission spectrum of the BGN and BGF glasses doped with 1 mol% of Tm_2_O_3_ in the range of the absorption bands of water.

Figure 2 shows the absorption spectra of the Tm^3+^ doped BGN and BGF samples under room temperature. All absorption bands belong to transition of Tm^3+^ ions from ground state to higher levels are labeled in Fig. 2. As expected, BGN and BGF samples have similar absorption peaks, and the ^3^H_6_-^1^G_4_ transition has not appeared, due to the UV cut-off wavelength of bismuthate glasses is redshift. Strong absorption around 790 nm indicates that these glasses can be excited efficiently by 808 nm LD. As shown in Fig. 3, BGF sample shows better IR transmittance than BGN sample. The absorption band ranging from 2700 to 3700 cm^−1^ is due to stretching vibrations of free OH^−^ groups. Hydroxyl and the fluorine ions are isoelectronic and their ionic size is similar^28^, hydroxyl ions can easily be removed by fluoride during melting through the reaction OH^−^ + F^−^ → HF + O^2−^. The OH^−^ absorption coefficient in the glass can be calculated by the IR transmission spectra, which is given by^31^


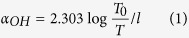


where *l* is the thickness of a sample, *T*_*0*_ and *T* are the transmission value of maximum and at 3000 cm^−1^, respectively. The OH^−^ absorption coefficient of BGN and BGF samples are calculated according to Eq. (1)cm^−1^ and 0.05 cm^−1^, respectively. It is obvious that typical OH^−^ groups’ absorption of BGN sample is much stronger than that of BGF sample at 3 μm regions, which is one of the main reasons for the difference between BGN and BGF samples in ~1.8 μm emission.

### Judd-Ofelt analysis

According to absorption spectra (Fig. 1), Judd-Ofelt (J-O) theory has been applied to determine the important spectroscopic and laser parameters of Tm^3+^ ion. In this paper, J-O intensity parameters *Ω*_*t*_ (*t* = 2, 4, and 6) are calculated and radiative transitions within 4*f* ^*n*^ configuration of Tm^3+^ is analyzed, the value of them list in Table 1. The value *Ω*_*2*_ of BGF are lower than those of BGN, however, they are still much larger than that of silicate^31^, tellurite^32^, fluoride^33^ and germanate^34^ glasses. As known *Ω*_*2*_ is related with the covalency between rare earth ions and ligands anions and reflects the asymmetry of local environment at Tm^3+^ site in the glass hosts. Large *Ω*_*2*_ means stronger covalency between the rare-earth ions and ligand anions, while the *Ω*_*6*_ has a relation with the overlap integrals of 4*f* and 5*d* orbits^26^. Large value of *Ω*_*6*_ exhibits the large value of emission bandwidth and spontaneous radiative probability of rare earth^31^. Values of *Ω*_*4*_ and *Ω*_*6*_ also provide some information on the rigidity and viscosity of hosts.

As shown in Table 2, spontaneous emission probability (*A*) for Tm^3+^ can also be calculated by using J-O theory, which is related with the J-O parameters and the refractive-index of host glass. Total spontaneous emission probability (∑*A*) of Tm^3+^: ^3^F_4_ level in BGN glass (454.8 s^−1^) is higher than that in BGF glass (406.38 s^−1^), so is the *A*_*rad*_ of transition Tm^3+^: ^3^H_4_ → ^3^F_4_. High *A* value in BGN suggests strong emission, especially the ~1.8 μm emission. Lower *A* and Higher *τ* in BGF are owing to the addition of fluoride could reduce the refractive-index and J-O parameters in bismuthate glass system. Compared with calculated radiative properties in germanate glasses^35^, BGN and BGF samples have higher *A*_*rad*_ value for each transition.

### Emission properties

**Figure 4:**
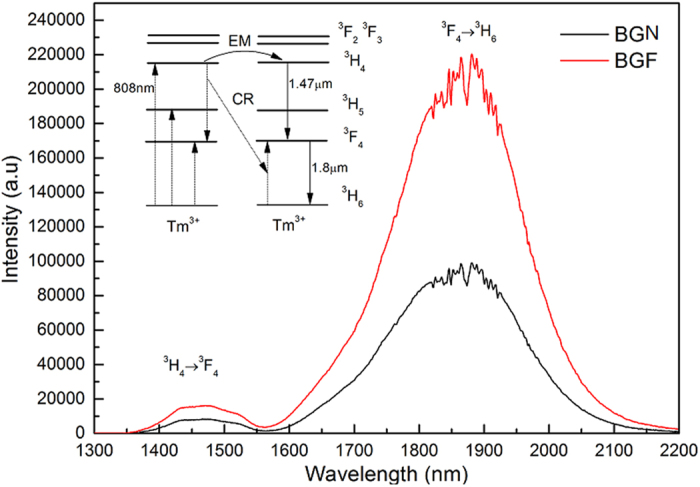
Room temperature ~1.8 μm emission spectra obtained by exciting with a cw laser diode at 808 nm for the BGN and BGF glasses doped with 1 mol% of Tm_2_O_3_. The inset is the energy level diagram and energy transfer sketch map of Tm^3+^ when pumped at 808 nm.

Figure 4 shows the ~1.47 μm and ~1.8 μm emission spectra in BGN and BGF samples under 808 LD pumped. After the BaF_2_ introduced, peak intensity of the ~1.8 μm emission in BGF is 2 times higher than that in BGN, while the intensity of ~1.47 emission is only a little change between two samples. As shown in the insert Fig. 4, the large intensity ratio of ~1800 nm to ~1470 nm (I_1800_/I_1470_) is related to the cross-relaxation (CR, ^3^H_6_ + ^3^H_4_ → ^3^F_4_ + ^3^F_4_)^36^.

**Figure 5:**
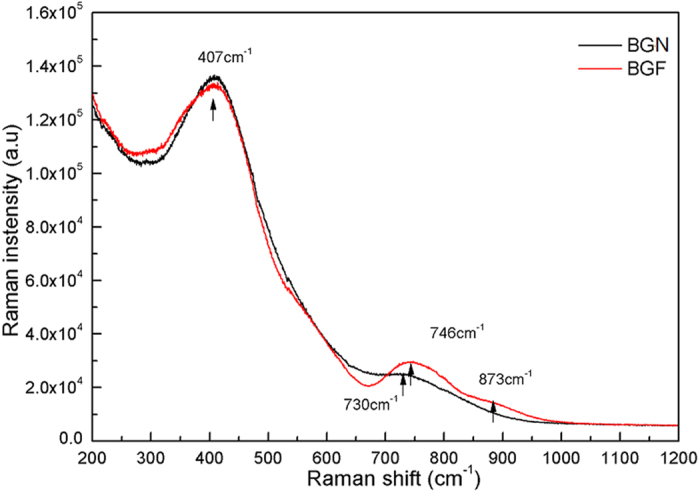
Raman spectra of BGN and BGF host glass samples in the range from 200 to 1200 cm^−1^.

With the introduction of BaF_2_, the maximum phonon energy of glass hosts lower accordingly, which can be seen from the measured Raman spectra shown in Fig. 5, the maximum phonon energy of BGN and BGF samples can be presumed about 746 cm^−1^ and 730 cm^−1^, respectively. The Raman scattering band higher than 700 cm^−1^ is mainly caused by the vibration of the tetrahedron group, the peak bond located in 756 cm^−1^ and 846 cm^−1^, correspond to the structure unit vibration of Ge-O and Ga-O, respectively^34^. For BGF sample, lower phonon energy is also a key factor for stronger ~1.8 μm emissions.

**Figure 6:**
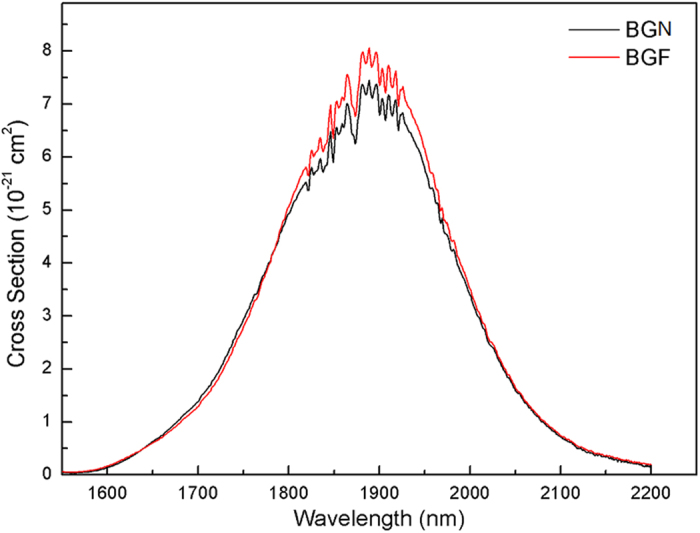
Stimulated emission cross-section of Tm^3+^:^3^F_4_→^3^H_6_ transition in BGN and BGF glasses doped with 1 mol% Tm_2_O_3_.

According to the Fuchtbauer-Ladenburg theory, ~1.8 μm emission cross section (*σ*_*em*_) is calculated^5^.


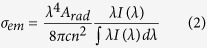


where *λ* is the wavelength, *A*_*rad*_ is the spontaneous emission probability calculated by J-O theory, *I*(*λ*) is the fluorescence intensity, *n* is the refractive index of the glass, and *c* is the light speed. It is noted that *σ*_*em*_ mainly related to ~1.8 μm emission spectrum and radiative transition probability of Tm^3+^: ^3^F_4_ → ^3^H_6_, which is a normalized line-shape function, respectively. According to Eq. (3), the stimulated emission cross-sections (*σ*_*em*_) of ~1800 nm calculated are shown in Fig. 6. It can be determined that *σ*_*em*_ of BGF sample performs a maximum 7.56 × 10^−21^ cm^2^ at 1865 nm, which is higher than that of BGN sample (7.01 × 10^−21^ cm^2^, centered at 1865 nm). For BGN and BGF samples, the values of the maximum stimulated emission cross-section at the wavelength of 1865 nm, which are larger than that of the fluorophosphate glasses^37^, silicate glasses^2^,^16^,^31^ and germanate glasses^38^, due to high refractive index, high J-O parameters and good emission, and are beneficial to ~1.8 μm laser action of Tm^3+^ ions.

The product of emission cross-section and radiative lifetime *σ*_*em*_ × *τ*_*rad*_ is an important parameter for laser materials to obtain high gain. As shown in Table 3, the calculated values *σ*_*em*_ × *τ*_*rad*_ of BGN and BGF samples are 15.42 × 10^−21^ cm^2^ ms and 18.59 × 10^−21^ cm^2^ ms, respectively, which are lower than silicate glass^31^ *σ*_*em*_ × *τ*_*rad*_= 28.48×10^−21^ cm^2^ ms. However, There are still larger than tellurite glass^24^ *σ*_*em*_ × *τ*_*rad*_ = 14.00 × 10^−21^ cm^2^ ms and germanate glasses^38^ *σ*_*em*_ × *τ*_*rad*_= 13.6 × 10^−21^ cm^2^ ms.

### Cross-relaxation process

Because of the cross-relaxation transfer process (^3^H_6_ + ^3^H_4_ → ^3^F_4_ + ^3^F_4_) is beneficial for the ~1800 nm emission^5^. It is necessary to study the cross-relaxation process between Tm^3+^ ions. According to the theory of Dexter and Forster, the cross-relaxation rate can be calculated by the integral overlap of absorption cross-sections and emission cross-sections^33^, which belongs to a dipole–dipole interaction. The microscopic transfer probability can be expressed by^34^


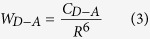


where *R* is the distance between donor and acceptor, *C*_*D−A*_ is the transfer constant defined as follows^15^ 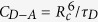, where *R*_*c*_ is the critical radius of the interaction and *τ*_*D*_ is the intrinsic lifetime of the donor-excited level. The transfer constant can be obtained according to Eq. (4) when phonons participate in the process to balance the energy gap^5^.


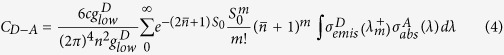


where *c* is the light speed, *n* is the refractive index, 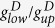 is the degeneracy of the lower and upper levels of the donor, respectively, 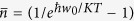 is the average occupancy of the phonon mode at temperature T, 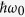 is the maximum phonon energy, *m* is the number of phonons that participate in the energy transfer, *S*_*0*_ is Huang–Rhys factor (0.31 for Tm^3+^)^5^, and 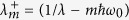 is the wavelength with *m* phonon creation. The caculated energy migration (EM, ^3^H_4_ + ^3^H_6_ → ^3^H_6_ + ^3^H_4_) and cross relaxation (CR, ^3^H_6_ + ^3^H_4_ → ^3^F_4_ + ^3^F_4_) processes in BG and BGF are listed in Table 4. Because of the transfer condition of *C*_*D−D*_ is much larger than *C*_*D−A*_, the hopping model is fulfilled in both BGN and BGF. to evaluate the energy transfer rate *W*_*ET*_^39^,


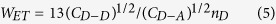


where *n*_*D*_ is the concentration of donor. According to Eq. (5), *W*_*ET*_ is calculated to be 938 cm^3^/s and 1020 cm^3^/s in BGN and BGF, respectively.

### Fluorescence lifetime

**Figure 7:**
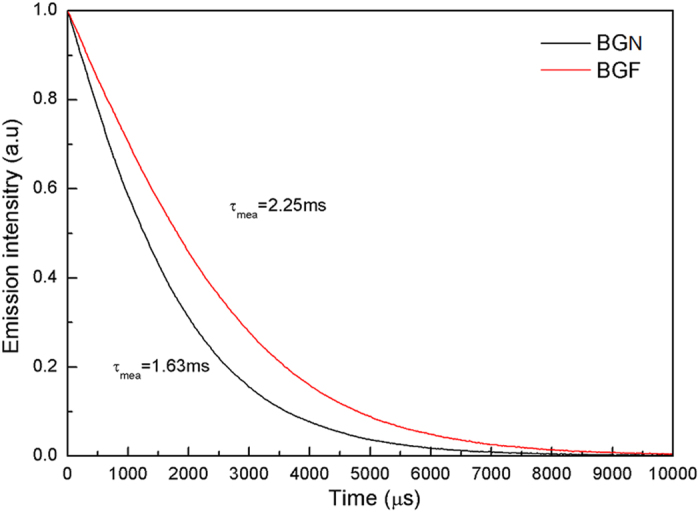
Luminescence decay curves of the ^3^F_4_ level of the Tm^3+^ ions obtained exciting resonantly the ^3^H_6_ → ^3^H_4_ absorption transition at 808 nm and monitoring the Tm^3+ 3^F_4_ → ^3^H_6_ emission at 1865 nm in BGN and BGF glasses doped with 1 mol% Tm_2_O_3_.

The fluorescence decays of the Tm^3+^: ^3^F_4_ level at room temperature is shown in Fig. 7. It can be seen that the measured lifetime *τ*_*mea*_ in BGN and BGF are 1.63 ms and 2.25 ms, respectively. The quantum efficiency (*η*) of the ^3^F_4_ → ^3^H_6_ emission can be calculated by


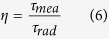


where *τ*_*mea*_ is the measured fluorescence lifetime and *τ*_*rad*_ is calculated with the Judd–Ofelt formalism. According to Eq. (6), the values of quantum efficiency for BGN and BGF are 74.09% and 91.46%, respectively, which are higher than silicate glass (13%)^31^, germanate glasss (55.52%)^5^, and lower than 70TeO_2_-20ZnO-10ZnF_2_ glass (164%)^40^. The larger radiative lifetime (*τ*_*mea*_) of Tm^3+^: ^3^F_4_ state is benefit for ~1.8 μm laser action. It can be seen that the measured lifetime is shorter than the calculated lifetime, due to nonradiative quenching^31^. The nonradiative decay caused from several mechanisms, such as energy transfer between the Tm^3+^ ions, multiphonon decay^29^, quenching by impurities (OH^−^), etc. The total rate of the ^3^F_4_ → ^3^H_6_ transition can be evaluated by^41^,^42^:


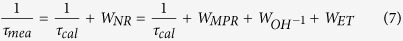


where 1*/τ*_*cal*_ is the spontaneous radiative probability *A*_*rad*_, *W*_*MPR*_ is the nonradiative multiphonon relaxation rate, *W*_*OH*_^−1^ is the nonradiative transition probability due to the energy transfer to OH^−^ impurities and *W*_*ET*_ represents an additional nonradiative loss mechanism due to the energy transfer between the RE ions. In this study, the concentrations of Tm^3+^ ions for BGN and BGF are the same, this third process can be neglected.

The multiphonon relaxation *W*_*MPR*_ can be expressed^43^:


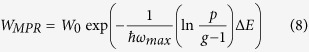


where *W*_*0*_ is an experimentally determined parameter which is independent of the particular RE ion. *∆E* is the energy gap between the ^3^F_4_ and ^3^H_6_ levels. *g* is the electron-phonon coupling strength parameter, and *ћω*_*max*_ is the highest phonon energy obtained from Raman spectra and *p* = *∆E/ћω*_*max*_. Multiphonon decay depends on the number of phonons required to bridge the energy gap to the next lower lying manifold. The higher the *ћω*_*max*_ is, the larger the multiphonon relaxation is.

*W*_*OH*_^*−*^ is proportional to the concentration of Tm^3+^ ions and the measured absorption coefficient of OH^−^ groups^42^,^44^. For BGF sample, after BaF_2_ introduced, the *α*_*OH*_^*−*^ shows a significantly decrease, *W*_*OH*_^−1^ is expected to decrease which results in a reduced nonradiative transition rate. Thus the lifetime is much longer while the quantum efficiency is higher in BGF. Generally, the relatively longer radiation lifetime is beneficial to reduce the laser oscillation threshold^45^.

## Conclusion

In conclusion, we reported on ~1.8 μm emission in Tm^3+^-doped Bi_2_O_3_-GeO_2_-Ga_2_O_3_ glasses in absence and presence of BaF_2_. The addition of BaF_2_ not only influences the network of glass, but also effectively reduces the content of hydroxyls and maximum phonon energy. For BGF sample, it shows a better thermal stability, and a stronger ~1.8 μm emission than that in BGN sample. It is also found that BGF glass possesses relatively large ~1.8 μm emission cross-section *σ*_*em*_ (7.56 × 10^−21^ cm^2^), measured fluorescence lifetime *τ*_*mea*_ (2.25 ms) and figure of merit gain *σ*_*em*_ × *τ*_*rad*_ (14.69 × 10^−21^ cm^2^ ms) corresponding to the Tm^3+^: ^3^F_4_ → ^3^H_6_ transition. Our results suggest that introduced the BaF_2_ into the glass network structure, which paves a way to enhance the ~1.8 μm emission properties and improve the fluorescence lifetime of Tm^3+^: ^3^F_4_ in Tm^3+^ doped bismuthate glass.

## Additional Information

**How to cite this article**: Han, K. *et al*. Optical characterization of Tm^3+^ doped Bi_2_O_3_-GeO_2_-Ga_2_O_3_ glasses in absence and presence of BaF_2_. *Sci. Rep.* **6**, 31207; doi: 10.1038/srep31207 (2016).

## Figures and Tables

**Table 1:** Judd-Ofelt intensity parameters in BGN and BGF samples.

Glass	Ω_*2*_ (×10^−20^ cm^2^)	Ω_*4*_ (×10^−20^ cm^2^)	Ω_*6*_ (×10^−20^ cm^2^)	Reference
BGN	6.26	0.24	1.6	This work
BGF	5.15	1.01	1.25	
silicate	3.40	0.46	0.66	16
Tellurite	3.20	2.01	1.83	29
Fluorophosphate	3.01	2.56	1.54	32
Germanate	3.93	1.1	1.1	35

**Table 2:** Calculated radiative properties in BGN and BGF samples.

Transition	λ (nm)	A_rad_ (s^−1^)	BGN sample				BGF sample		
			∑A (s^−1^)	β (%)	τ (ms)	A_rad_ (s^−1^)	∑A (s^−1^)	β (%)	τ (ms)
^**3**^**F**_**4**_ **→** ^**3**^**H**_**6**_	1847	454.48	454.48	100	2.20	406.38	406.38	100.00	2.46
^**3**^**H**_**5**_ **→** ^**3**^**F**_**4**_	3563	27.5	609.08	4.53	1.64	21.45	507.51	4.23	1.97
**→**^**3**^**H**_**6**_	1216	581.51		95.47		486.07		95.77	
^**3**^**H**_**4**_ **→** ^**3**^**H**_**5**_	2428	10.81	3679.83	0.29	0.27	26.49	2894.29	0.92	0.35
**→**^**3**^**F**_**4**_	1444	283.61		7.71		235.88		8.15	
**→**^**3**^**H**_**6**_	810	3385.40		92.00		2631.92		90.93	
^**3**^**F**_**3**_ **→** ^**3**^**H**_**4**_	5200	7.05	4938.24	0.14	0.20	7.00	4302.28	0.16	0.23
**→**^**3**^**H**_**5**_	1655	832.61		16.86		685.69		15.94	
**→**^**3**^**F**_**4**_	1310	184.22		3.73		133.42		3.10	
**→**^**3**^**H**_**6**_	701	3914.36		79.27		3476.17		80.80	
^**3**^**F**_**2**_ **→** ^**3**^**F**_**3**_	20449	0.01	2155.96	0.00	0.46	0.01	1639.24	0.00	0.61
**→**^**3**^**H**_**4**_	4145	41.78		1.94		33.83		2.06	
**→**^**3**^**H**_**5**_	1531	370.17		17.17		347.88		21.22	
**→**^**3**^**F**_**4**_	10722	2.09		0.10		1.63		0.10	
**→**^**3**^**H**_**6**_	678	1741.92		80.80		1255.88		76.61	
^**1**^**G**_**4**_ **→** ^**3**^**F**_**2**_	1632	20.61	5445.72	0.38	0.18	24.28	4493.39	0.54	0.22
**→**^**3**^**F**_**3**_	1511	118.63		2.18		97.46		2.17	
**→**^**3**^**H**_**4**_	1171	714.61		13.12		533.45		11.87	
**→**^**3**^**H**_**5**_	790	1946.18		35.74		1434.64		31.93	
**→**^**3**^**F**_**4**_	647	383.41		7.04		318.74		7.09	
**→**^**3**^**H**_**6**_	479	2262.29		41.54		2084.82		46.40	
^**1**^**D**_**2**_ **→** ^**1**^**G**_**4**_	1538	432.58	71946.23	0.60	0.01	374.52	62466.60	0.60	0.02
**→**^**3**^**F**_**2**_	792	1258.84		1.75		1569.68		2.51	
**→**^**3**^**F**_**3**_	762	3113.90		4.33		2523.72		4.04	
**→**^**3**^**H**_**4**_	665	5208.87		7.24		3930.55		6.29	
**→**^**3**^**H**_**5**_	522	244.90		0.34		188.45		0.30	
**→**^**3**^**F**_**4**_	455	55623.17		77.31		43031.91		68.89	
**→**^**3**^**H**_**6**_	365	6063.96		8.43		10847.76		17.37	
^**1**^**I**_**6**_ **→** ^**1**^**D**_**2**_	1424	0.00	25009.80	0.00	0.04	0.00	26885.62	0.00	0.04
**→**^**1**^**G**_**4**_	739	3182.74		12.73		3497.78		13.01	
**→**^**3**^**F**_**2**_	509	2202.92		8.81		1707.47		6.35	
**→**^**3**^**F**_**3**_	497	55.70		0.22		49.53		0.18	
**→**^**3**^**H**_**4**_	453	3851.15		15.40		4264.61		15.86	
**→**^**3**^**H**_**5**_	382	150.15		0.60		125.55		0.47	
**→**^**3**^**F**_**4**_	345	14686.53		58.72		15939.71		59.29	
**→**^**3**^**H**_**6**_	291	880.60		3.52		1300.96		4.84	

∑*A* is the total spontaneous emission probability of each level, *A*_*rad*_ is the spontaneous emission probability of each transition, *τ* is the calculated radiative lifetime.

**Table 3:** Calculated emission cross-sections σ_em_, radiative lifetime *τ*_*rad*_, and σ_em_ × *τ*_*rad*_ of Tm^3+^: ^3^F_4_ → ^3^H_6_ in BGN and BGF samples.

Glass	σ_em_ (×10^−21^ cm^2^)	*τ*_*rad*_ (ms)	σ_em_ × *τ*_*rad*_ (×10^−21^ cm^2^ ms)	Reference
BGN	7.01	2.25	15.42	This work
BGF	7.56	2.46	18.59	
silicate	3.6	7.91	28.48	28
Tellurite	8.1	1.73	14.00	24
Germanate	7.7	1.77	13.60	37

**Table 4:** Energy transfer parameters of the energy migration and cross-relaxation processes in BGN and BGF samples.

Glass	EM		CR		W_ET_(10^−20^ cm^3^/s)
	M% phonons	*C*_*D−D*_ (10^−40^ cm^6^/s)	M% phonons	*C*_*D−A*_ (10^−40^ cm^6^/s)	
BGN	0, 1	35.4	0, 1, 2	16.0	938
	99.99, 0.01		12.78, 83.99, 3.23		
BGF	0, 1	37.8	0, 1, 2	18.6	1020
	99.99, 0.01		16.07, 79.37, 4.56		
